# Sound Touch Viscosity (STVi) for Thyroid Gland Evaluation in Healthy Individuals: A Pilot Study

**DOI:** 10.2174/0115734056335791241202115022

**Published:** 2025-01-02

**Authors:** Feng Mao, Yuemingming Jiang, Yunzhong Wang, Zhenbin Xu, Zhuo Wei, Xueli Zhu, Libin Chen, Shengmin Zhang

**Affiliations:** 1 Department of Medical Ultrasound, The First Affiliated Hospital of Ningbo University, Ningbo 315010, China; 2 Ningbo University School of Medicine, Ningbo 315010, China

**Keywords:** Viscosity, Ultrasound, Thyroid gland, Healthy subjects, Thyroid elastography, linear array transducer

## Abstract

**Objective:**

This prospective study aimed to establish the typical viscosity range of the thyroid gland in healthy individuals using a new method called the Sound Touch Viscosity (STVi) technique with a linear array transducer.

**Methods:**

Seventy-eight healthy volunteers were enrolled between March, 2023 and April, 2023. Thyroid viscosity was measured using the Resona R9 ultrasound system equipped with a linear array transducer (L15-3WU). Each patient had three valid viscosity measurements taken for each thyroid lobe, and the average values were analyzed. Thyroid gland stiffness was measured and analyzed simultaneously.

**Results:**

The study included 51 women and 27 men with an average age of 48 years. The mean viscosity measurement for a normal thyroid gland was 1.10 ± 0.41 Pa.s (ranging from 0.38 to 2.25 Pa.s). There were no significant differences in viscosity between the left and right lobes of the thyroid gland. We found no significant variations in viscosity based on gender, age, or body mass index (BMI). There was a notable positive correlation between thyroid viscosity and stiffness measurements (*r* = 0.717, *p* < 0.001).

**Conclusion:**

Our findings suggest that STVi is a highly reliable method for assessing the thyroid. This technique holds promise as a new, non-invasive approach to evaluating thyroid parenchyma viscosity.

## 1 INTRODUCTION

Shear Wave Elastography (SWE) is a reliable method used in medical imaging to measure tissue stiffness non-invasively [1-3]. Current SWE techniques assume that tissues are uniformly elastic and isotropic; that is, the shear wave propagation velocity is independent of the shear wave frequency [4, 5]. However, biological tissues contain not only a considerable number of protein fibres with elasticity but also a significant amount of matrix and extracellular fluid with viscosity, which represents the ability to resist deformation and generate internal friction when a force is applied. Thus, biological tissues possess intrinsic viscoelastic properties rather than pure elasticity [6]. Studies have demonstrated that shear wave propagation is affected not only by tissue elasticity, which determines shear wave velocity but also by tissue viscosity, which causes shear wave dispersion [7]. This means that the relationship between the force applied by acoustic radiation and the resulting strain is both time-dependent and nonlinear due to the presence of both elasticity and viscosity [8, 9]. Neglecting tissue viscosity can lead to inaccuracies in elasticity measurements and potentially bias crucial diagnostic information [5, 7].

Research on viscosity as an imaging parameter is still in its early stages. Shear wave dispersion, which involves the frequency-dependent changes in shear wave velocity and attenuation, can serve as an indirect method for assessing viscosity [7, 10]. Previous studies have indicated that increases in necrosis and inflammatory changes in soft tissues correlate with higher dispersion, suggesting elevated tissue viscosity [11, 12]. Consequently, viscosity assessment holds promise for detecting inflammatory and fibrotic alterations in thyroid tissue [13]. Prior literature has provided a range of values for normal thyroid parenchyma viscosity [14, 15]. However, these studies utilized curvilinear transducers, which may not accurately characterize surface structures. Recently, the Sound Touch Viscosity (STVi) module in the Resona R9 system, developed by Shenzhen Mindray BioMedical Electronics Co., Ltd., has enabled the measurement of thyroid viscoelasticity. The calculation is based on SWE imaging, whereby the shear wave velocity at different frequencies is extracted and calculated. Subsequently, the viscosity coefficient is determined using a viscoelasticity fitting model [16]. This module offers visualization and quantitative data on tissue viscosity using a high-frequency linear array transducer in a two-dimensional imaging mode. Viscosity measurements are conducted locally at specific points of interest within an organ, with results expressed in Pascal seconds (Pa.s).

To date, there are limited clinical studies on thyroid viscosity, all of which employed convex array transducers to assess normal thyroid viscosity values [14, 15]. However, given that the thyroid is a superficial organ, a higher frequency transducer is necessary to accurately assess parenchymal structure changes. Currently, there is a lack of research on thyroid viscosity measurement using linear array transducers suitable for thyroid scans. Thus, the objective of this study was to establish reference values for normal thyroid viscosity using linear array transducers in a healthy cohort.

## 2 METHODS

### 2.1 Study Population

A prospective monocentric study was carried out at a tertiary hospital from March, 2023 to April, 2023, involving 78 healthy individuals. Participants were selected from patients undergoing neck sonography for non-thyroid issues, with normal levels of thyroid-stimulating hormone (TSH) ranging from 0.3 to 5.0 mIU/L, no family history of thyroid conditions, and normal thyroid parenchyma appearance on sonography. Patients with a history of thyroid disorders (such as autoimmune diseases, inflammation, or thyroid nodules), thyroid dysfunction, or prior radioactive iodine treatment were excluded.

Ethical approval for this study was obtained from the local ethics committee of the First Affiliated Hospital of Ningbo University, ensuring adherence with the 2000 revision of the World Medical Association's Declaration of Helsinki in Edinburgh. Written consent was obtained from all participants prior to the study's commencement.

### 2.2 Viscosity and Stiffness Measurements

Viscosity and stiffness measurements of thyroid parenchyma were conducted using the STVi module of the Resona R9 Ultrasound system (Shenzhen Mindray BioMedical Electronics, Co., Ltd., Shenzhen, China). All examinations were performed by a radiologist (JYMM) with 17 years of experience in sonography and 5 years of experience in SWE, who was blinded to the subjects’ clinical data.

The STVi mode analyzes shear wave propagation speed across various frequencies to assess shear wave dispersion within soft tissues. Viscosity measurement is obtained simultaneously with stiffness measurement as the STVi mode is integrated with the SWE mode.

The subjects were positioned supine with their heads slightly tilted backward to expose and flatten the front of the neck adequately. Initial assessment of the thyroid parenchyma was conducted using a high-frequency linear array transducer (L15-3WU) to evaluate thyroid size and overall appearance.

To conduct viscosity and stiffness measurements of each thyroid lobe, the same transducer was employed in the sagittal plane. The transducer was positioned vertically and securely on the skin surface, with an adequate amount of gel applied to prevent pre-pressurization. Upon achieving a clear acoustic window for the thyroid parenchyma, the viscoelastic mode was activated. The acquisition box size was set to the instrument’s default, ensuring it encompassed the entire thyroid lobe. Participants were instructed to momentarily refrain from breathing or swallowing. Image acquisition commenced when the motion stability index (M-STB index), located in the upper right corner of the display, indicated 4 or 5 green stars, signifying minimal external motion and ensuring reliable measurements. The M-STB index employs a five-star scale, where 1 to 3 stars indicate that the measurement should not proceed due to external motion, as it would result in erroneous values. Conversely, 4 to 5 stars indicate that measurements should proceed as no external motion is detected.

**Fig. (1):**
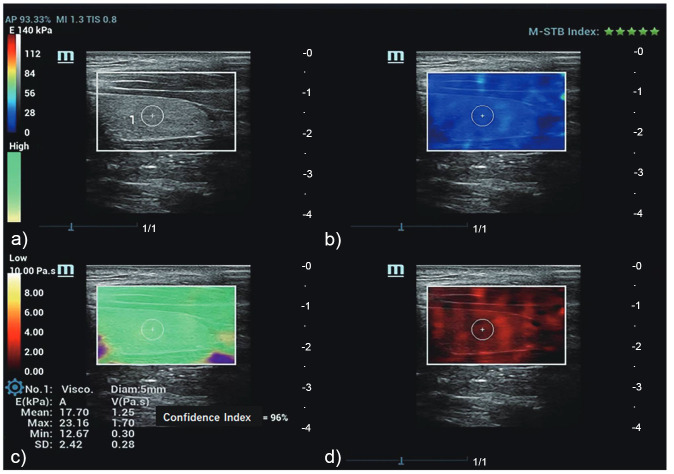
Viscosity and stiffness measurements performed in the left thyroid lobe of a healthy subject. **a**) Conventional ultrasound map displays the left thyroid parenchyma without pathologies. **b**) A 2D SWE map illustrates the elasticity distribution within the acquisition box (range 0 to 140 kPa), with blue indicating softer tissue and red indicating harder tissue. **c**) Quality control map indicates the homogeneity of the acquisition box, with green areas in the reliability map denoting samples without artifacts and purple areas identifying samples with artifacts. The confidence index is 96%. **d**) The viscosity map demonstrates the viscosity distribution within the acquisition box (range 0 to 10 Pa.s), with red indicating low viscosity values and yellow-white indicating high viscosity values. The quantitative viscosity and elasticity results are displayed in the lower-left corner of the image. The ROI measurements display the mean viscosity and stiffness values of the left thyroid lobe as 1.25 Pa.s and 17.7 kPa, respectively.

After image acquisition, a quad-view mode was presented (Fig. **1**) using the grayscale map, SWE map, quality control map, and viscosity map. The grayscale map provided a clear representation of the thyroid parenchyma structure. The SWE map utilized color-coded representation to depict two-dimensional tissue stiffness evaluation, with colors ranging from dark blue to yellow to dark red. These colors corresponded to Young's modulus values (kPa), where blue indicated soft tissue and red indicated increased stiffness. The quality control map displayed a confidence index and a reliability map, indicating homogeneity in shear wave intensity within the acquisition box. Green areas in the reliability map indicated artifact-free samples, while purple areas identified samples with artifacts. The viscosity map utilized color coding, where yellow-white denoted high viscosity and red represented low viscosity values. The instrument was configured to provide viscosity values from 0.00 to 10.00 Pa.s and stiffness values from 0.00 to 140.00 kPa.

For measurement, the diameter of the region of interest (ROI) was set between 3 and 5 mm. A valid measurement was defined as measurements obtained with a confidence index of over 90%, and the ROI was positioned in the green area as indicated by the quality control map. The mean stiffness values (kPa) and viscosity values (Pa.s) for the ROI measurement were both displayed in the lower-left corner of the image.

Image acquisition was repeated three times for each thyroid lobe to acquire three valid viscosity and stiffness measurements. The mean of these three measurements was utilized to evaluate glandular viscosity and stiffness for further analysis.

To assess intra- and inter-observer reliability, a subgroup of 30 subjects from our study were randomly selected prospectively. The mean viscosity measurements for each subject in this subgroup were independently obtained by a second radiologist (WYZ) following the aforementioned protocol, who was blinded to the subjects’ clinical data and previous image acquisition. This second radiologist had 8 years of experience in sonography and 2 years of experience in SWE.

### 2.3 Statistical Analysis

Statistical analyses were carried out using MedCalc Version 20 (MedCalc Software Corp., Brunswick, ME, USA). The distribution of numerical variables was assessed using the Kolmogorov–Smirnov test. Normally distributed continuous variables were presented as mean ± SD, while skewed distributed variables were expressed as median and range interval. Categorical variables were presented as percentages and numbers. The mean differences between subgroups were compared using either the Student t test or the Mann-Whitney U test, with the exception of subgroups based on age distribution, which were assessed using one-way analysis of variance (ANOVA) or Kruskal-Wallis H test. The upper and lower endpoints covering 95% of the reference interval of viscosity measurements were determined along with their respective 90% confidence intervals (CI) using the robust method. Pearson’s correlation coefficient was employed to evaluate correlations between normally distributed data, while Spearman’s rank correlation (rho) was used to assess correlation between non-normally distributed data. Inter-observer and intra-observer agreement for viscosity were assessed using the intraclass correlation coefficient (ICC). Statistical significance for all tests was set at *p* < 0.05.

## 3 RESULTS

Our study included a total of 78 healthy volunteers, with 51 women (65.4%) and 27 men (34.6%). The mean age ± SD in the entire group was 48 ± 13 years. Their mean body mass index (BMI) was 24.42 ± 4.21 kg/m^2^. All of them (100%) had valid measurements.

**Fig. (2):**
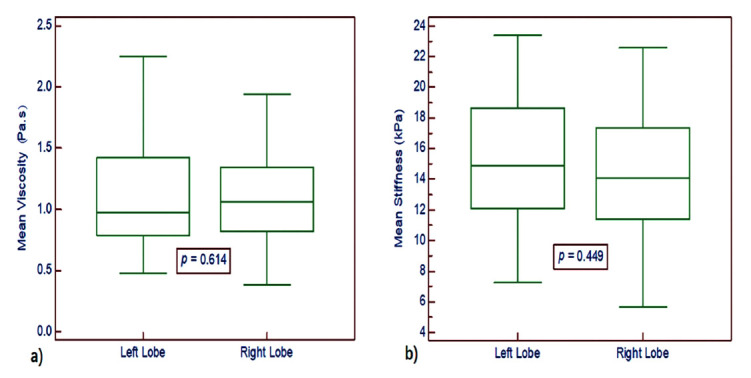
Box and whisker plot comparing mean viscosity and stiffness values for the right and left thyroid lobes. **a**) Comparison of mean viscosity values. **b**) Comparison of mean stiffness values.

Comparisons were made between the mean viscosity and stiffness values of both the right and left thyroid lobes for each subject (mean of 3 values for each lobe). The mean viscosity and stiffness measurements for the left thyroid lobe were 1.13 ± 0.46 Pa.s (range, 0.48 - 2.25 Pa.s) and 15.21 ± 4.03 kPa (range, 7.24 - 23.37 kPa), respectively. For the right lobe, the values were 1.09 ± 0.38 Pa.s (range, 0.38 - 1.94 Pa.s) and 14.49 ± 4.11 kPa (range, 5.66 - 22.58 kPa), respectively. No significant differences were observed between viscosity and stiffness values of the left and right thyroid lobes (Fig. **2**). Therefore, the mean measurements of both lobes of the thyroid gland for each healthy subject were included in subsequent analyses.

**Fig. (3):**
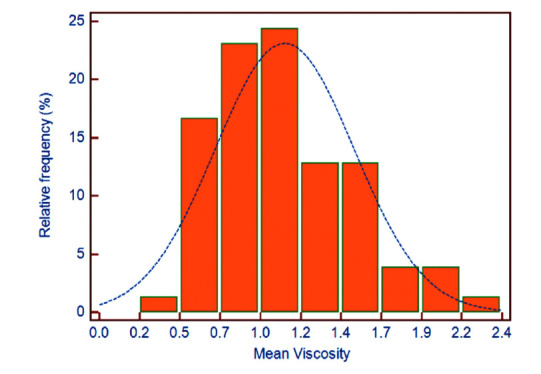
Distribution of mean thyroid viscosity values in healthy subjects, ranging from 0.38 to 2.25 Pa.s.

Mean viscosity and stiffness values for both lobes of thyroid parenchyma are presented in Table **1**. Normal thyroid mean viscosity values ranged between 0.38 and 2.25 Pa.s (Fig. **3**). The 95% reference interval of viscosity measurements ranged from 0.22 (90% CI: 0.09 to 0.35) to 1.91 (90% CI: 1.73 to 2.06) Pa.s.

No statistically significant differences were observed in mean viscosity and stiffness values between males and females (*p* = 0.833; *p* = 0.868, respectively) (Table **2**).

The cohort of healthy subjects was divided into three age-based groups: young adults (18-39 years), midlife adults (40-59 years), and older adults (60-74 years). The group of older adults exhibited the highest viscosity values, while the group of midlife adults demonstrated the lowest values. Nevertheless, no statistically significant differences were identified between the age groups in relation to these values (*p* = 0.518). Furthermore, no statistically significant differences were observed between the age groups in terms of stiffness values (*p* = 0.699) (Table **3**).

In accordance with the criteria set forth by the BMI scale, the cohort of healthy subjects was classified into two distinct groups: individuals with a normal weight (BMI range of 18.5–24.9) and those with a higher BMI (≥25.0), indicating overweight. There were no significant differences found between the two groups in terms of viscosity values (1.17 ± 0.44 Pa.s *vs*. 1.05 ± 0.38 Pa.s (*p* = 0.198)) or stiffness values (15.05 ± 4.36 kPa *vs*. 14.54 ± 3.85 kPa (*p* = 0.587)) (Table **4**).

**Fig. (4):**
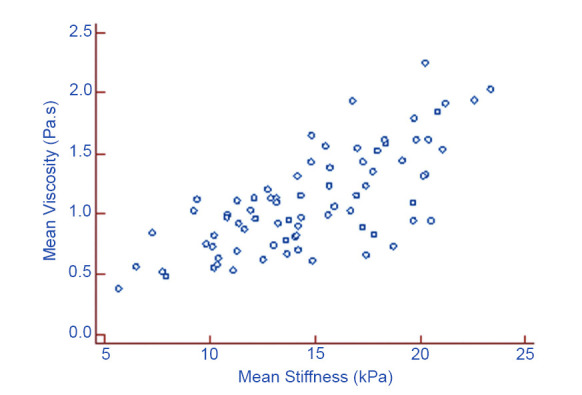
Scatter diagram depicting the correlation between mean viscosity values and stiffness values of thyroid parenchyma in healthy subjects.

A significant positive correlation was observed between mean viscosity values and stiffness values in the thyroid parenchyma (*r* = 0.717; *p* < 0.001) (Fig. **4**). Possible correlations between mean viscosity values and age and BMI were also examined, but no statistical significance was found (*r* = 0.033, *p* = 0.774; *r* = 0.026, *p* = 0.823).

Intra-observer and inter-observer variability testing was conducted for mean viscosity assessments of the thyroid, revealing high reproducibility; the ICC had a value of 0.84 (95% CI: 0.70 - 0.92) for intra-observer agreement and 0.80 (95% CI: 0.61 - 0.90) for inter-observer agreement.

## 4 DISCUSSION

There is growing evidence suggesting that combining viscosity algorithms with shear wave dispersion modules can serve as a supplementary tool for assessing soft tissue pathologies [17, 18]. In a study by Sugimoto *et al*. [12], it was noted that the dispersion slope, which reflects viscosity, is more effective than shear wave speed in predicting the extent of necroinflammation in the liver. Therefore, viscosity may provide additional insights into the pathological and physiological changes in thyroid parenchyma, whether diffuse or focal. Rianna *et al*. [19] investigated the viscoelastic properties of the thyroid in experimental models and discovered that malignant thyroid cells maintained consistent viscoelastic properties regardless of substrate stiffness. In contrast, normal thyroid cells exhibited increased stiffness with higher substrate stiffness, demonstrating a similar trend in dynamic viscosity. This distinction could potentially aid in distinguishing between cancerous and normal cells.

To date, there have been limited studies on the implementation of this innovative ultrasound technology in thyroid clinical assessment. Previous studies have prospectively examined the potential of Viscosity plane-wave UltraSound (ViPLUS) technology to establish viscosity reference values for the healthy human thyroid [14, 15]. However, this technology was initially designed for liver evaluation and only incorporated viscosity modules compatible with convex array transducers. Recently, Mindray has developed a viscosity algorithm specifically tailored for linear array transducers, particularly targeting superficial organs, such as the breast and thyroid. Utilizing a high-frequency linear array transducer offers the advantage of accurately characterizing fine structures in superficial tissues, facilitating precise localization of target areas. Moreover, linear array transducers demonstrate less variability in detecting shear wave motion compared to convex array transducers at shallow depths [20]. The initial step in validating this novel approach is to establish a representative viscosity value for soft tissue, which could potentially assist in the early detection of thyroid abnormalities.

To our knowledge, this study represents the first attempt to establish a reference value for thyroid viscosity in healthy subjects using a linear transducer. When novel technologies are introduced, assessing their feasibility is crucial to determine their clinical applicability. Stoian *et al*. [14] conducted a prospective clinical study on 121 patients, demonstrating a feasibility of 95.1% in using a convex array transducer equipped with ViPLUS technology to measure thyroid viscosity. Our study indicates that employing a linear array transducer with STVi technology for thyroid viscosity measurement is highly feasible, with a feasibility of 100%. Furthermore, both intra- and inter-observer reproducibility were deemed good, underscoring the reproducibility of the STVi technique for viscosity assessment.

Our study found no significant difference in viscosity and stiffness values between the left and right thyroid lobes. Consequently, the average viscosity and stiffness values of thyroid parenchyma in healthy subjects were determined to be 1.10 ± 0.41 Pa.s and 14.77 ± 4.07 kPa, respectively. In comparison to previously reported values [14, 15], the mean viscosity value of the thyroid in our study was lower, potentially attributable to the different algorithms employed in our measurements with a linear array transducer compared to previous studies utilizing convex array transducers [21]. The mean stiffness value in our study was similar to the results reported by Sebag *et al*. [22] and Bhatia *et al*. [23] but lower than the 19.5 ± 7.6 kPa reported by Vlad *et al*. [24]. Variability in thyroid parenchyma stiffness may be influenced by various technical factors, including vendor-specific implementation of shear wave elastography and the increase in compression of the thyroid gland, also known as pre-load [19].

In our study, no significant differences in thyroid viscosity values were observed based on gender, age, or BMI, which is consistent with the findings reported by Stoian *et al*. [14]. Petea-Balea *et al*. [15] also reported similar results, although they noted that the viscosity and stiffness values of normal-weight subjects were significantly lower than those of overweight subjects. These findings suggest that normal thyroid viscosity values, akin to stiffness values, exhibit relative stability across these demographic parameters, which may be attributed to the inherent biomechanical properties of the thyroid tissue. It is important to note that while these factors may influence other physiological parameters, they do not seem to substantially alter the viscosity of thyroid parenchyma. This consistency could be due to the fact that viscosity is primarily a function of the tissue's internal friction and resistance to deformation, which are less likely to be influenced by external demographic variables.

A strong positive correlation was identified between thyroid stiffness and viscosity values. Although viscosity and elasticity represent distinct tissue properties, they are both rooted in the physics of shear wave propagation, with viscosity influenced by shear wave dispersion and elasticity influenced by shear wave speed [7]. The correlation observed in our study may be attributed to these shared mechanisms. Similar findings have been reported not only in studies focusing on the thyroid [14, 15] but also in research involving organs, such as the liver, kidney, and peripheral muscles [12, 25, 26]. The positive correlation between thyroid stiffness and viscosity values emphasizes the necessity of considering a range of tissue properties when assessing organ health. This approach can facilitate a more comprehensive understanding of tissue mechanics. Therefore, future research should investigate the underlying biological and biomechanical factors that contribute to this relationship, with the aim of developing improved diagnostic and therapeutic strategies for various diseases affecting these organs.

A notable limitation of our study is the relatively small sample size for analysis. Therefore, future studies should involve larger sample sizes to enhance the validity of the findings. Additionally, there is a gender distribution imbalance among our study participants, with a predominance of female subjects. Nonetheless, this study demonstrated no significant differences in mean viscosity values of normal thyroid between males and females.

## CONCLUSION

In this pilot study, we employed a linear array transducer with STVi technology to ascertain the typical viscosity range of thyroid parenchyma in healthy individuals. This represents a novel and promising non-invasive method for assessing the viscoelasticity of thyroid parenchyma. The data obtained on thyroid viscosity in this study may serve as a reference for viscosity evaluation in future studies involving patients with thyroid pathologies.

## Figures and Tables

**Table 1:** Mean viscosity and stiffness values of thyroid parenchyma in healthy subjects.

-	-	Value
**Viscosity (Pa.s)**	Mean±SD	1.10±0.41
-	95% CI	1.01-1.20
**Stiffness (kPa)**	Mean±SD	14.77±4.07
-	95% CI	13.85-15.69

**Abbreviations: **CI = confidence interval, SD = standard deviation.

**Table 2:** Mean viscosity and stiffness values of thyroid tissue in a group of healthy subjects according to gender.

-	-	Male (n=27)	Female (n=51)	p-value
**Viscosity (Pa.s)**	Mean±SD	1.09±0.48	1.11±0.38	0.833
-	95% CI	0.90-1.28	1.00-1.22	-
**Stiffness (kPa)**	Mean±SD	14.67±4.70	14.83±3.75	0.868
-	95% CI	12.81-16.52	13.77-15.88	-

**Abbreviations: **CI = confidence interval, SD = standard deviation.

**Table 3:** Mean viscosity and stiffness values of the thyroid parenchyma in healthy subjects group based on the age distribution.

-	-	Young Adults	Midlife Adults	Older Adults	p-value
-	-	(18-39, n=23)	(40-59, n=36)	(60-74, n=19)	
**Viscosity**	Mean±SD	1.14±0.42	1.05±0.34	1.17±0.53	0.518
**(Pa.s)**	95% CI	0.96-1.32	0.93-1.16	0.91-1.42	-
**Stiffness**	Mean±SD	14.29±4.04	14.76±3.55	15.37±5.07	0.699
**(kPa)**	95% CI	12.55-16.04	13.56-15.96	12.93-17.82	-

**Abbreviations: **CI = confidence interval, SD = standard deviation.

**Table 4:** Mean viscosity and stiffness values of the thyroid parenchyma in healthy subjects group according to BMI.

-	-	Normal Weight	Overweight	p-value
-	-	(BMI=18.5–24.9, n=43)	(BMI≥25.0, n=35)	
**Viscosity**	Mean±SD	1.05±0.38	1.17±0.44	0.198
**(Pa.s)**	95% CI	0.93-1.17	1.02-1.32	-
**Stiffness**	Mean±SD	14.54±3.85	15.05±4.36	0.587
**(kPa)**	95% CI	13.36-15.73	13.55-16.55	-

**Abbreviations: **BMI = body mass index, CI = confidence interval, SD = standard deviation.

## Data Availability

All data generated or analyzed during this study are included in this published article.

## LIST OF ABBREVIATIONS

BMI: Body Mass Index
CI: Confidence Interval
ICC: Intraclass Correlation Coefficient
M-STB index: Motion Stability Index
ROI: Region of Interest
SD: Standard Deviation
STVi: Sound Touch Viscosity
SWE: Shear Wave Elastography
TSH: Thyroid-stimulating Hormone
ViPLUS: Viscosity Plane-Wave UltraSound

## STANDARDS OF REPORTING

STROBE guidelines were followed.
